# Supplementary material for: Phenolic Acids and Flavonoids from Pithecellobium dulce (Robx.) Benth Leaves Exhibit Ovicidal Activity against Haemonchus contortus
Source: Plants (Basel). 2022 Sep 28;11(19):2555. doi: 10.3390/plants11192555 (PMC9573131; doi:10.3390/plants11192555)
Supplement: Supplementary file 1 [file plants-11-02555-s001.zip › plants-1910681-supplementary.pdf]

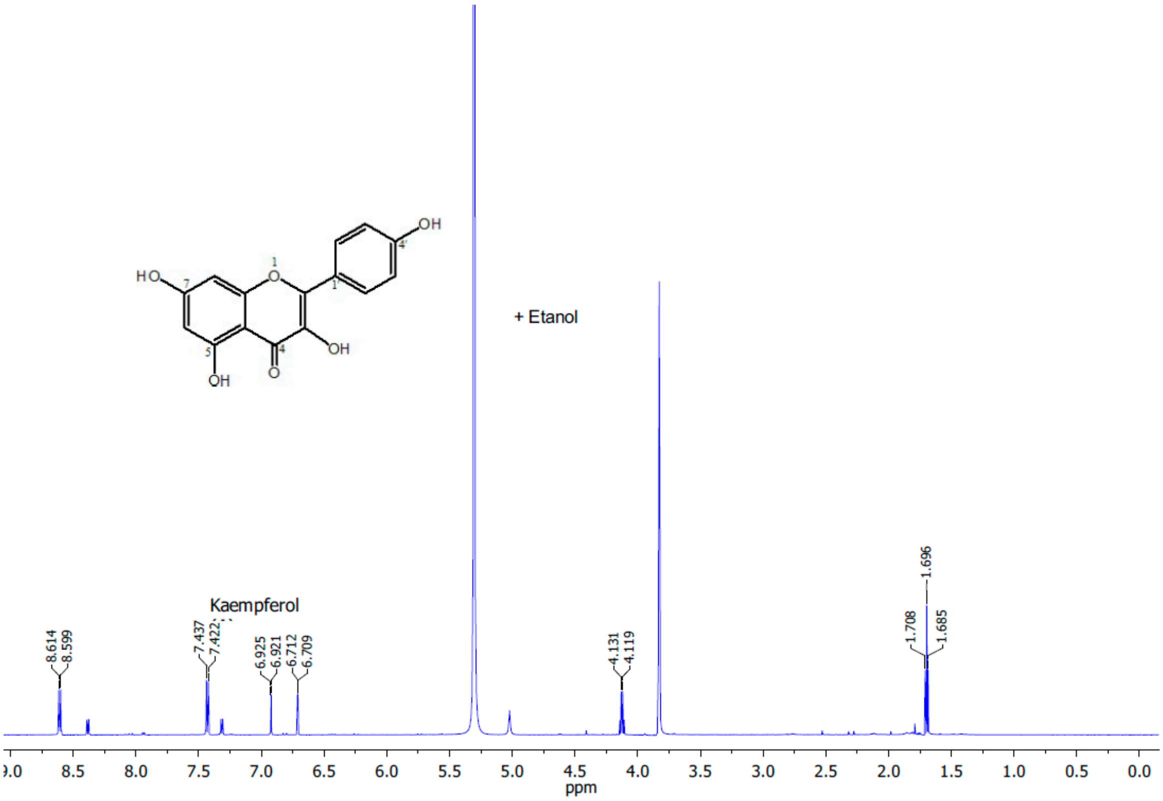

Figure S1. 1H NMR (CD3OD, 600 MHz) of Kaempferol (1)

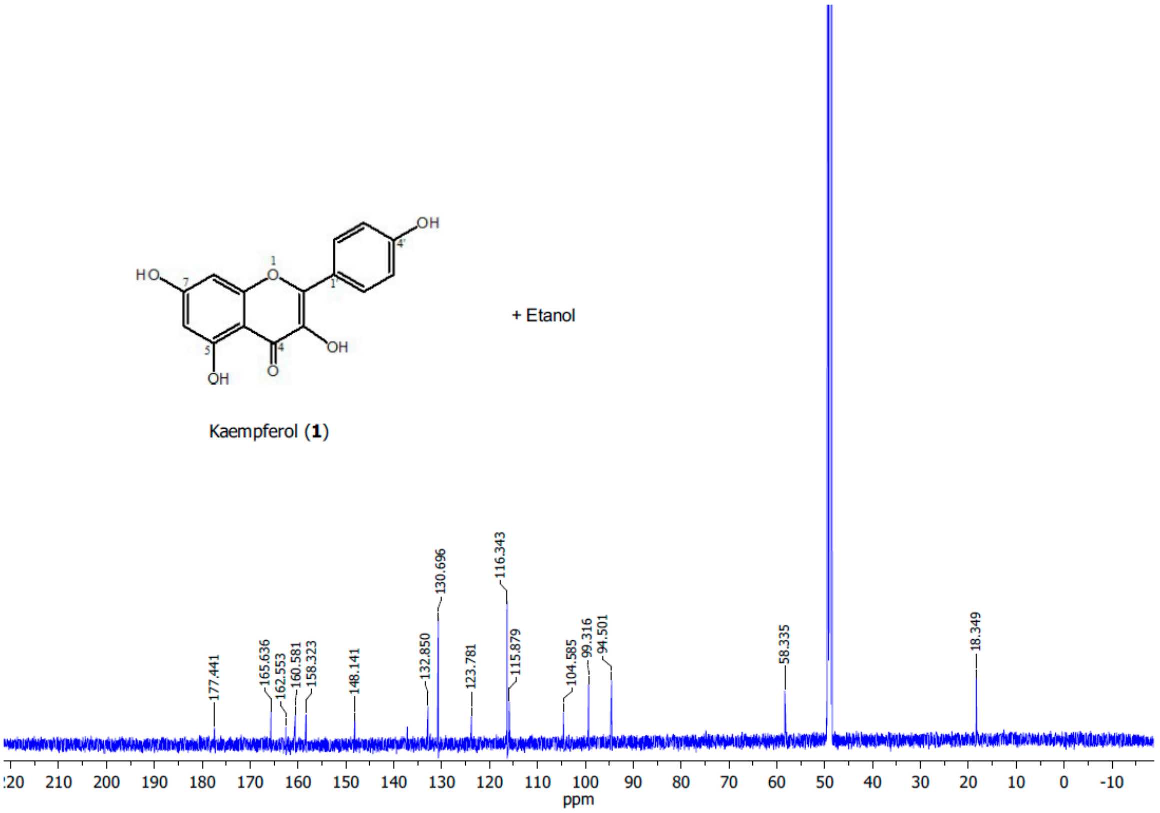

Figure S2. 13C NMR (CD3OD, 150 MHz) of Kaempferol (1)

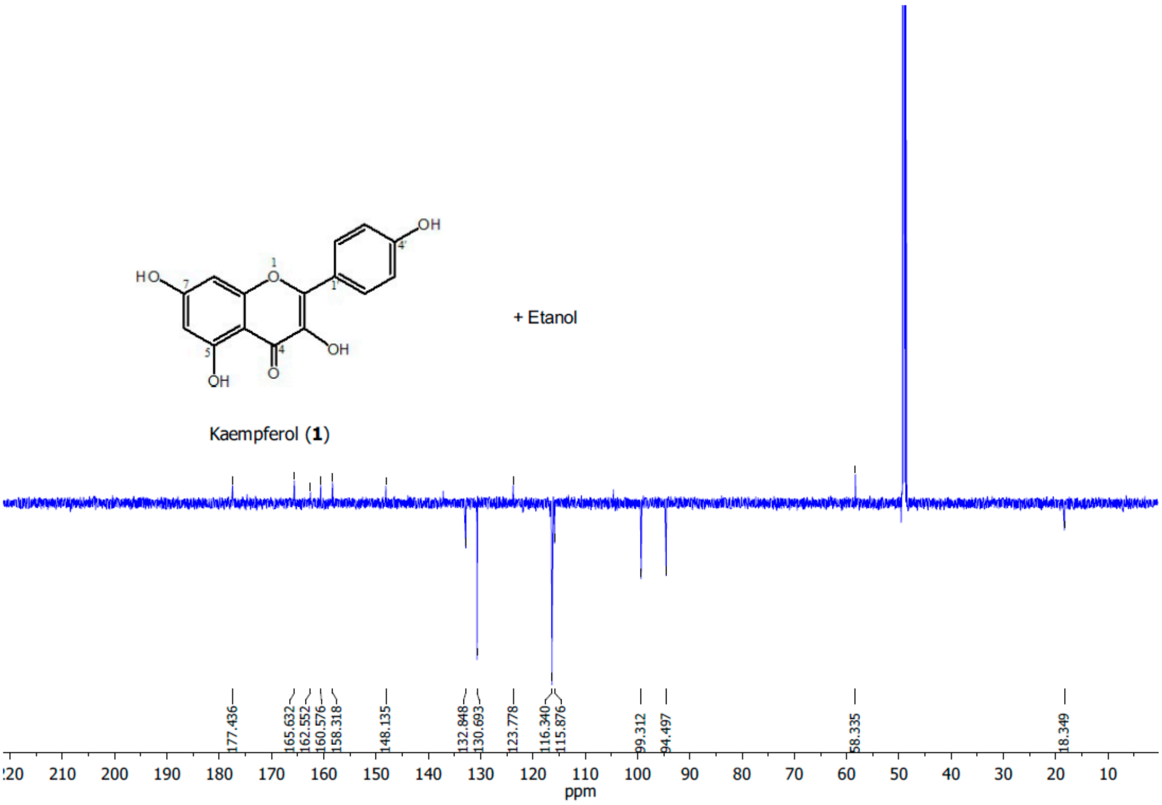

Figure S3. 13C (DEPT) NMR (CD3OD, 150 MHz) of Kaempferol (1)

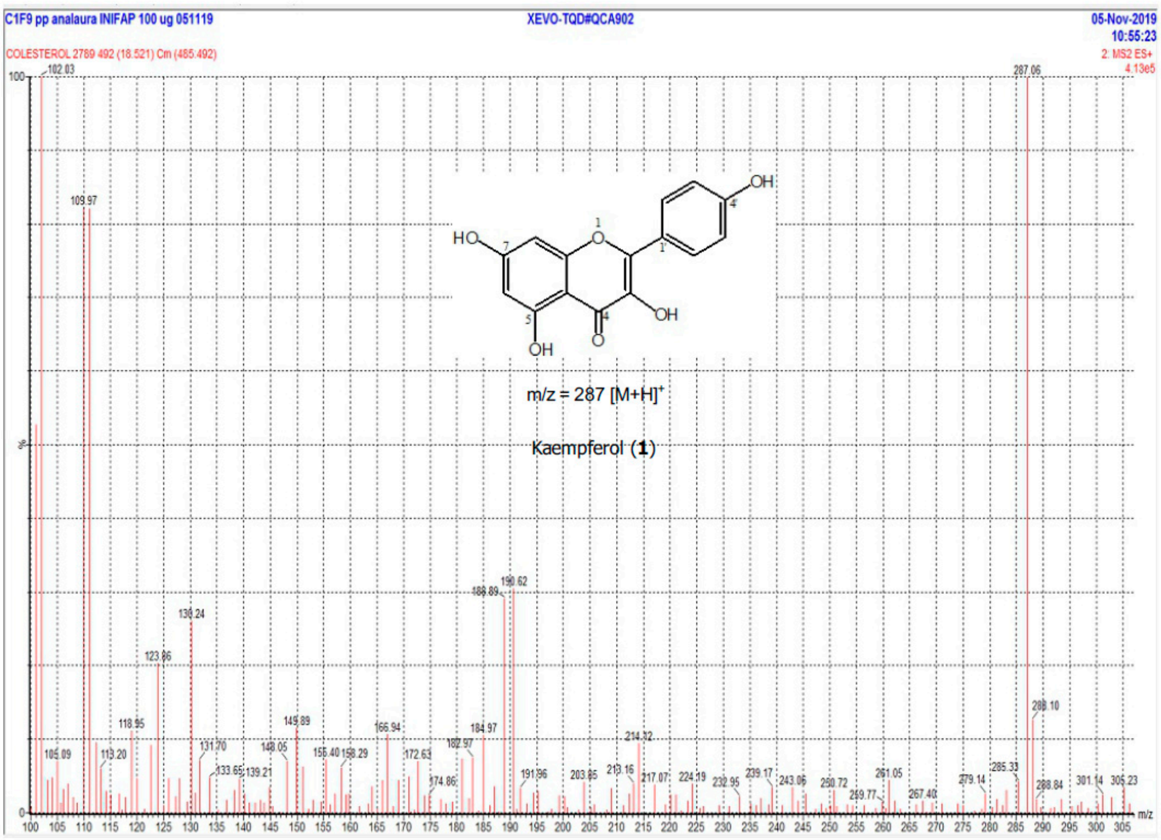

Figure S4. Mass spectrum(MS) of Kaempferol (1)

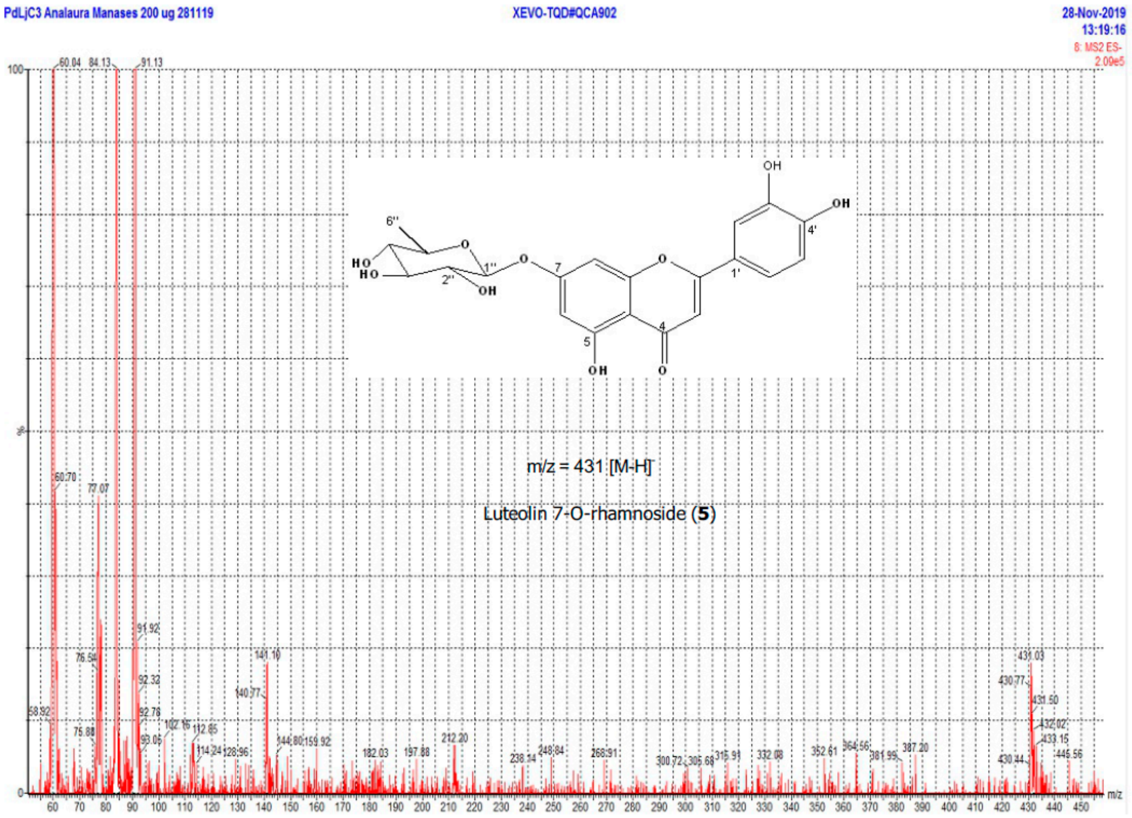

Figure S5. Mass spectrum(MS) of Luteolin 7-O-rhamnoside (5)

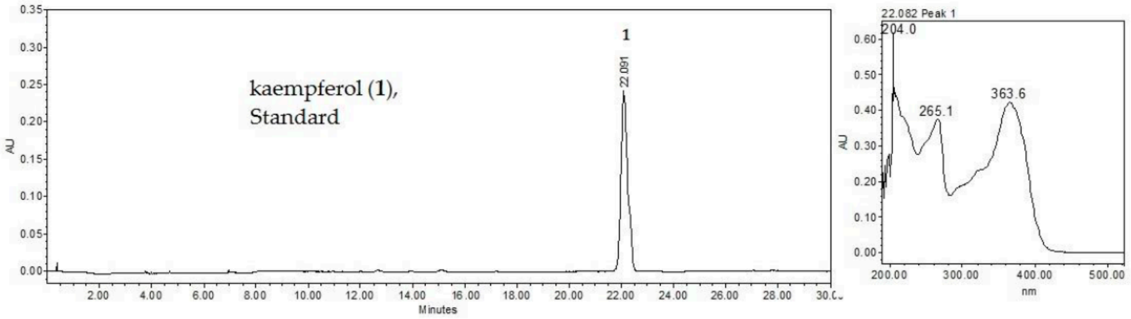

Figure S6. HPLC chromatogram and UV spectrum of Kaempferol (1) standard

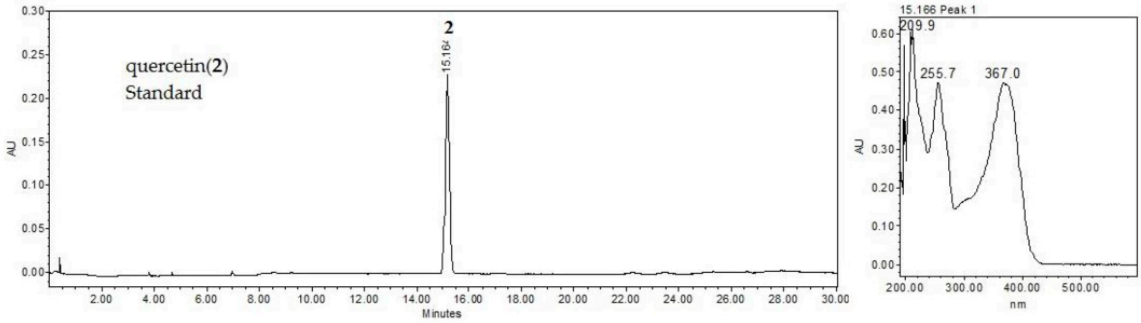

Figure S7. HPLC chromatogram and UV spectrum of Quercetin (**2**) standard

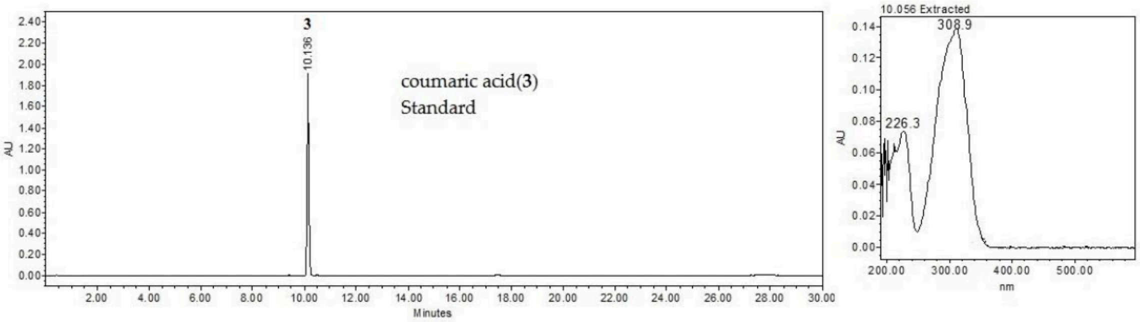

Figure S8. HPLC chromatogram and UV spectrum of Coumaric acid (3) standard

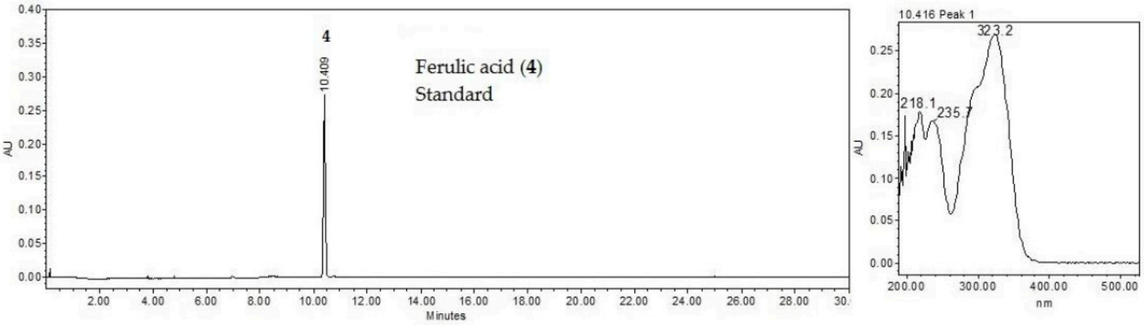

Figure S9. HPLC chromatogram and UV spectrum of Ferulic acid (4) standard

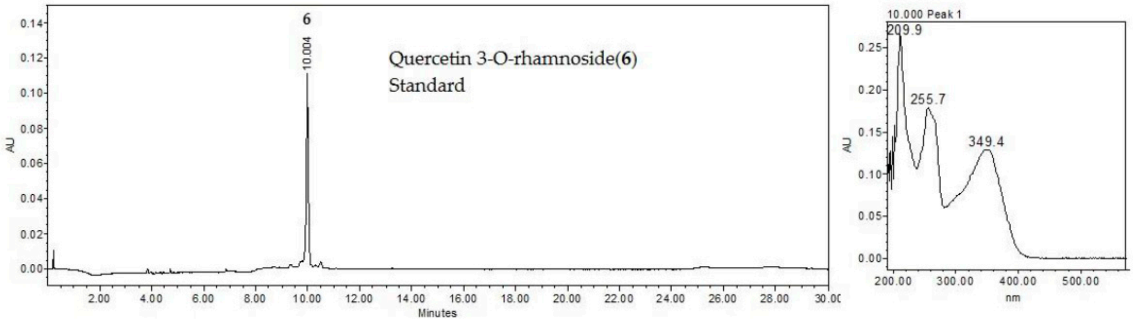

Figure S10. HPLC chromatogram and UV spectrum of quercetin 3-O-rhamnoside (6) standard

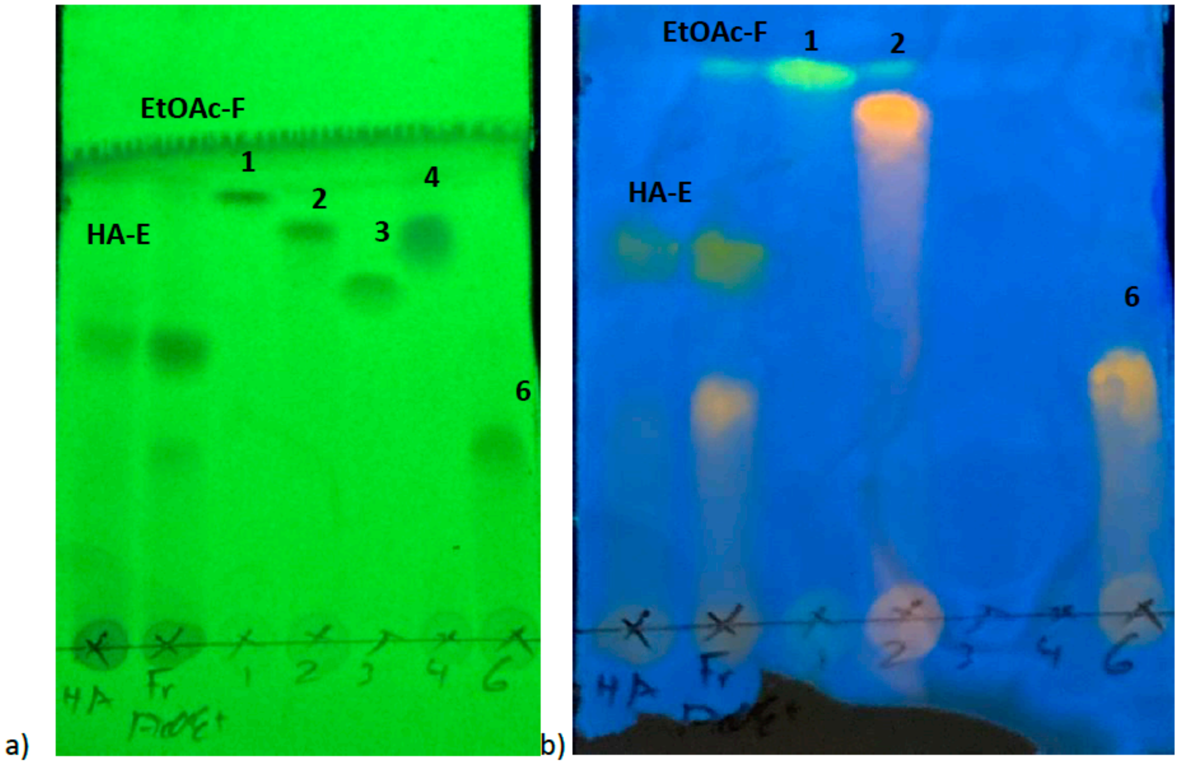

Figure S11. Normal phase TLC of the hydroalcoholic extract (HA-E) and the ethyl acetate fraction (EtOAc-F) compared to compounds 1-4 and 6 (standards). Elution system 85:15 dichloromethane:methanol, a) UV lamp (366 nm) before being developed and b) revealed with flavonoids (254 nm).

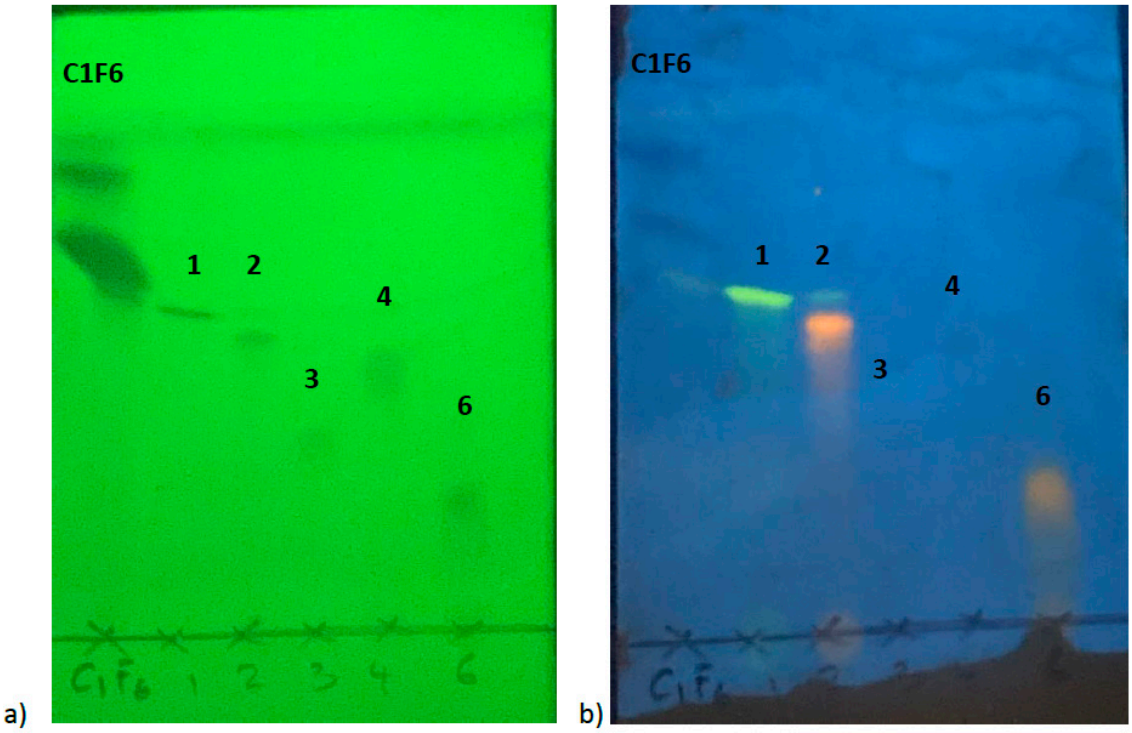

Figure S12. Normal phase TLC of fraction (C1F6) compared to compounds 1-4 and 6 (standards). Elution system 85:15 dichloromethane:methanol, a) UV lamp (366 nm) before

being developed and b) revealed with flavonoids (254 nm).

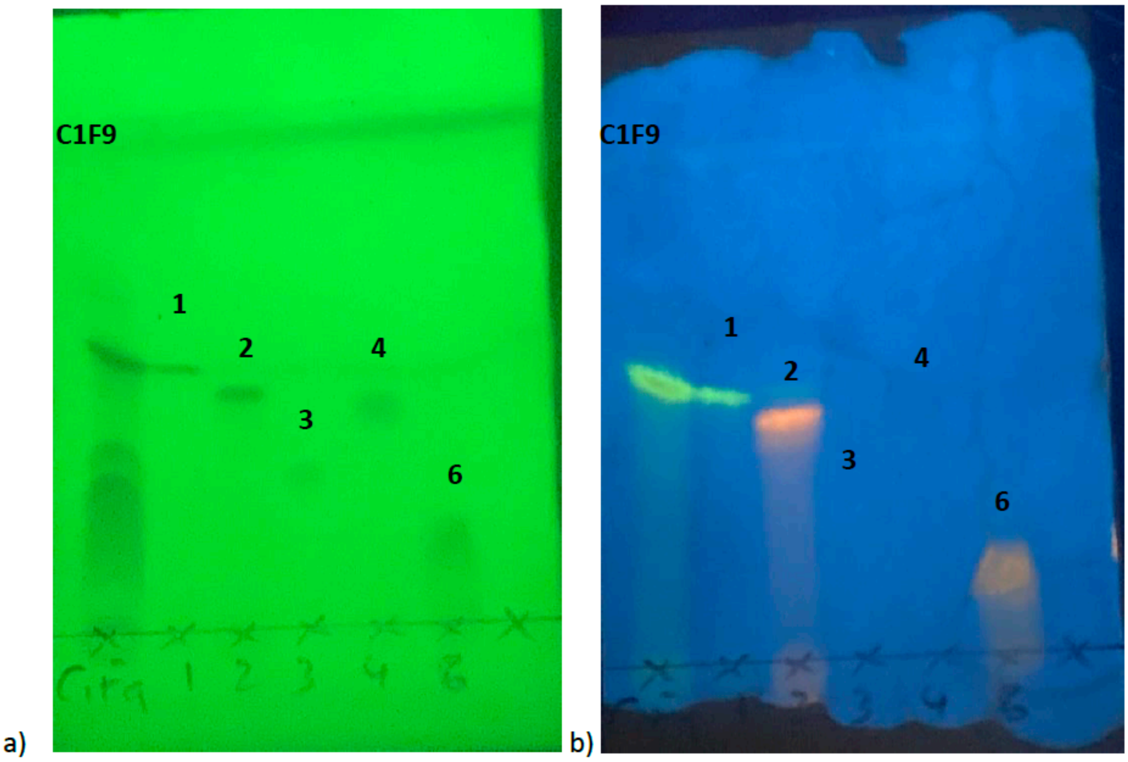

Figure S13. Normal phase TLC of fraction (C1F9) compared to compounds 1-4 and 6 (standards). Elution system 85:15 dichloromethane:methanol, a) UV lamp (366 nm) before being developed and b) revealed with flavonoids (254 nm).

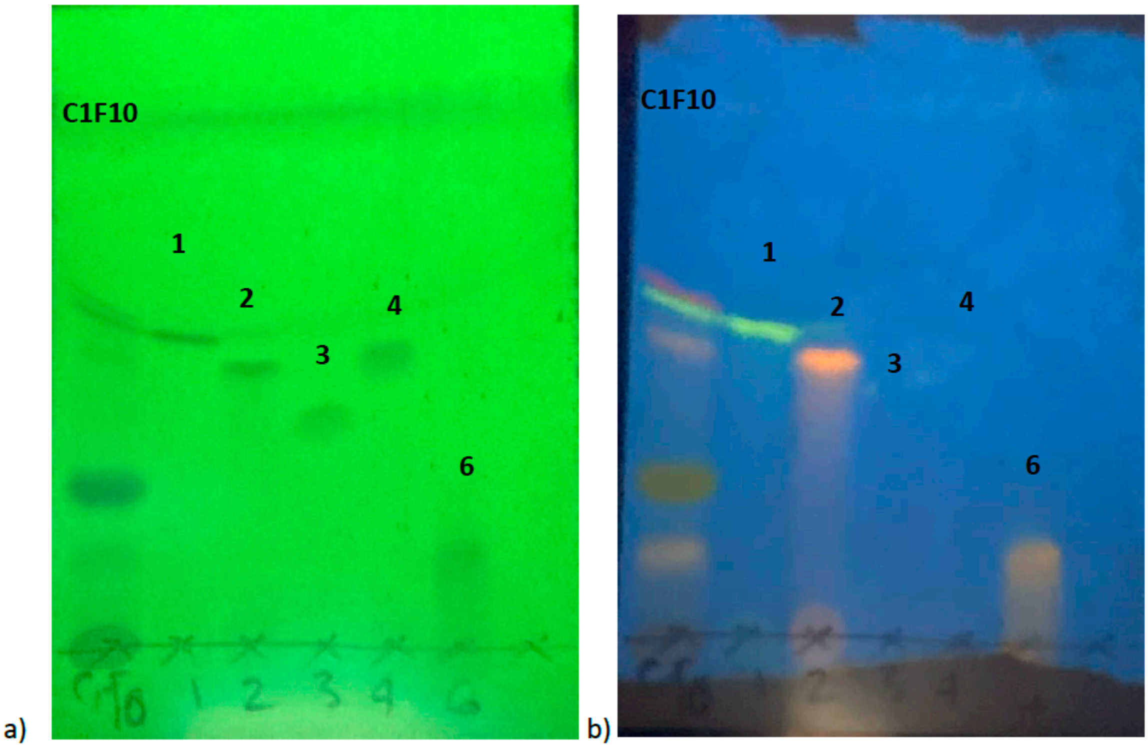

Figure S14. Normal phase TLC of fraction (C1F10) compared to compounds 1-4 and 6 (standards). Elution system 85:15 dichloromethane:methanol, a) UV lamp (366 nm) before

being developed and b) revealed with flavonoids (254 nm).

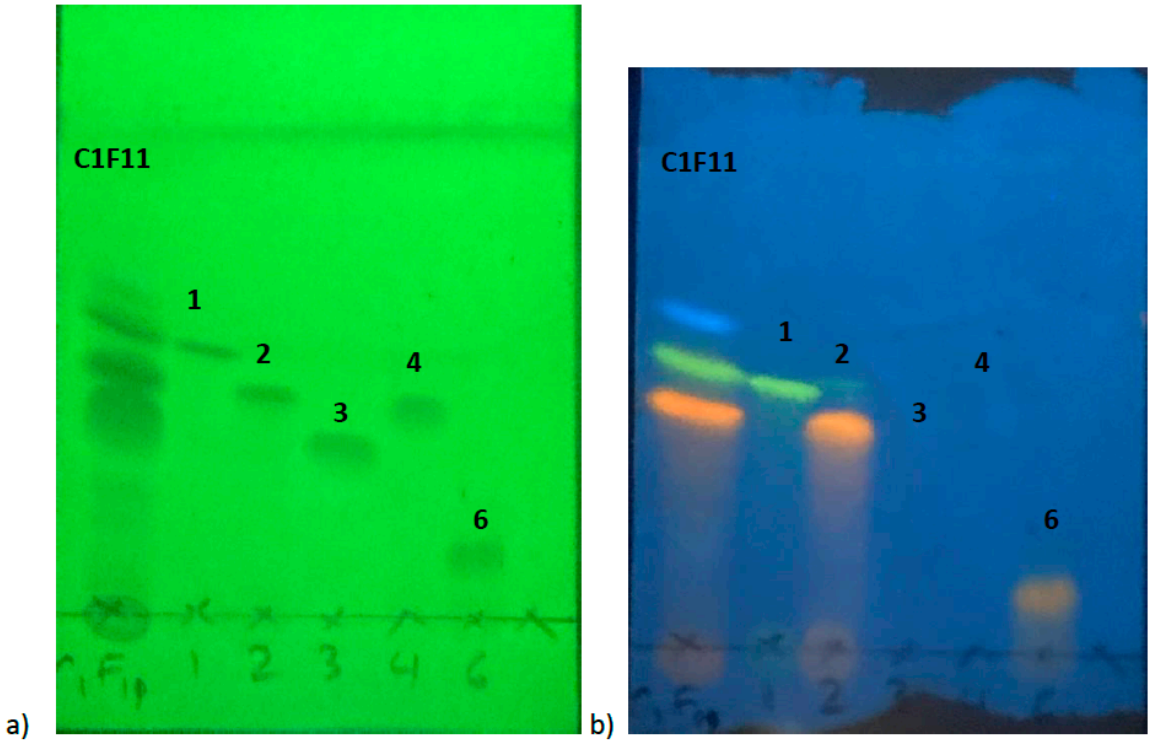

Figure S15. Normal phase TLC of fraction (C1F11) compared to compounds 1-4 and 6 (standards). Elution system 85:15 dichloromethane:methanol, a) UV lamp (366 nm) before being developed and b) revealed with flavonoids (254 nm).

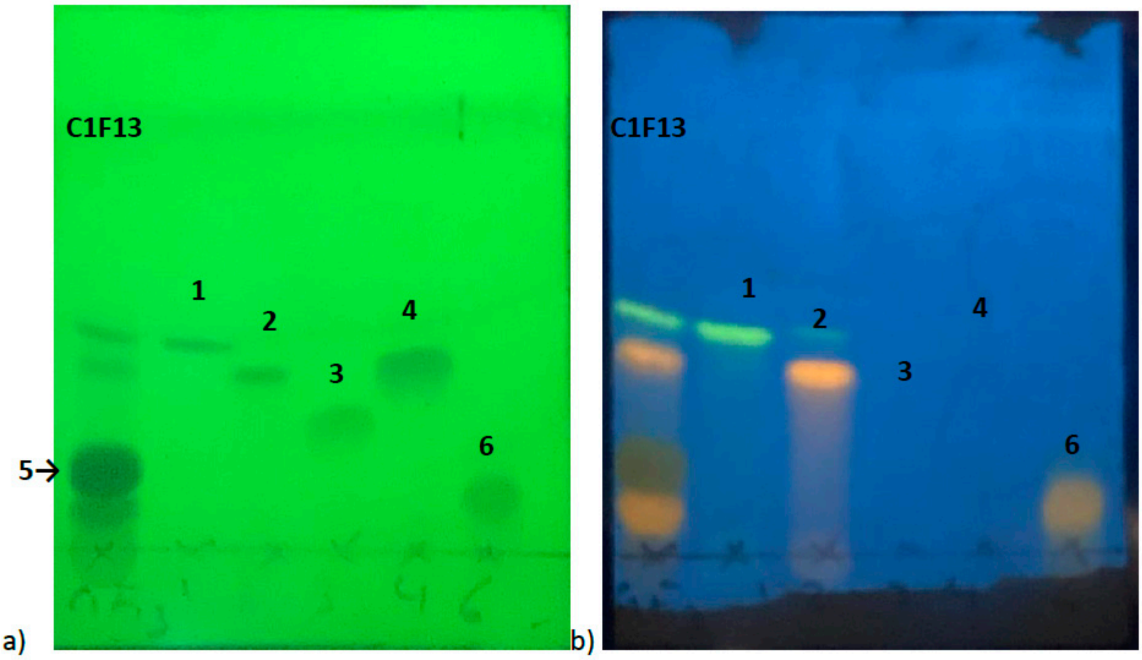

Figure S16. Normal phase TLC of fraction (C1F13) compared to compounds 1-4 and 6 (standards). Elution system 85:15 dichloromethane:methanol, a) UV lamp (366 nm) before being developed and b) revealed with flavonoids (254 nm).

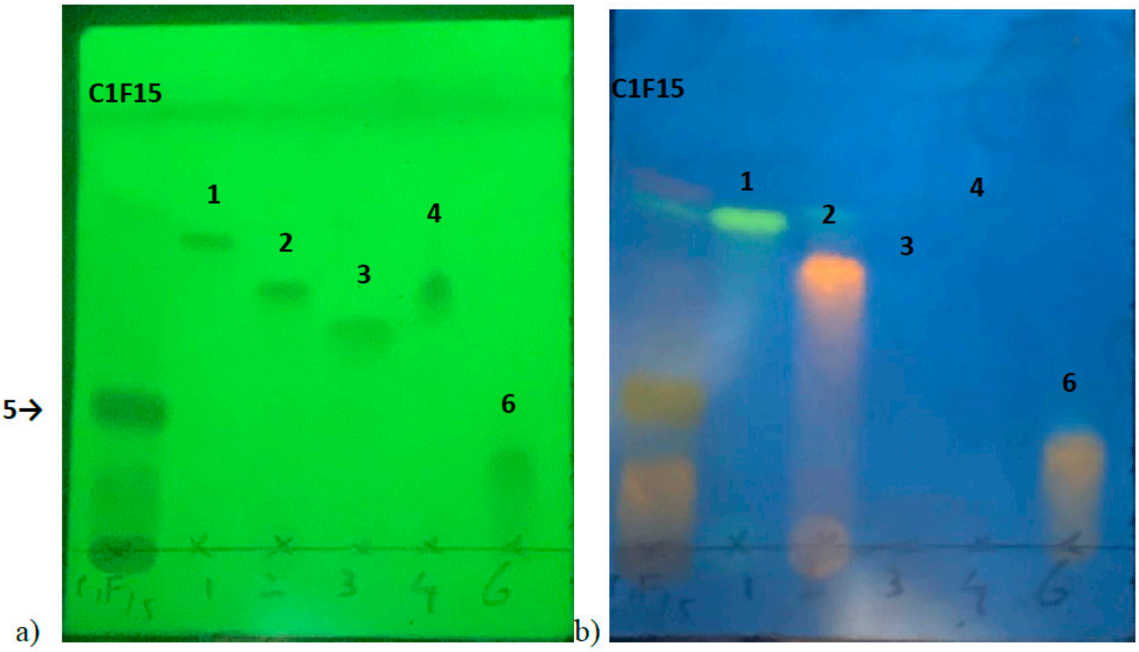

Figure S17. Normal phase TLC of fraction (C1F15) compared to compounds 1-4 and 6 (standards). Elution system 85:15 dichloromethane:methanol, a) UV lamp (366 nm) before being developed and b) revealed with flavonoids (254 nm).
